# Neutrophil extracellular traps-associated modification patterns depict the tumor microenvironment, precision immunotherapy, and prognosis of clear cell renal cell carcinoma

**DOI:** 10.3389/fonc.2022.1094248

**Published:** 2022-12-22

**Authors:** Zhi-Hai Teng, Wen-Ce Li, Zhi-Chao Li, Ya-Xuan Wang, Zhen-Wei Han, Yan-Ping Zhang

**Affiliations:** Department of Urology, The Second Hospital of Hebei Medical University, Shijiazhuang, China

**Keywords:** neutrophil extracellular traps, ccRCC, subtypes, prognosis, immune tumor environment

## Abstract

**Background:**

Neutrophil extracellular traps (NETs) are web-like structures formed by neutrophils, and their main function is antimicrobial defense. Moreover, NETs have numerous roles in the pathogenesis and progression of cancers. However, the potential roles of NET-related genes in renal cell carcinoma remain unclear. In this study, we comprehensively investigated the NETs patterns and their relationships with tumor environment (TME), clinicopathological features, prognosis, and prediction of therapeutic benefits in the clear cell renal cell carcinoma (ccRCC) cohort.

**Methods:**

We obtained the gene expression profiles, clinical characteristics, and somatic mutations of patients with ccRCC from The Cancer Genome Atlas database (TCGA), Gene Expression Omnibus (GEO), and ArrayExpress datasets, respectively. ConsensusCluster was performed to identify the NET clusters. The tumor environment scores were evaluated by the “ESTIMATE,” “CIBERSORT,” and ssGSEA methods. The differential analysis was performed by the “limma” R package. The NET-scores were constructed based on the differentially expressed genes (DEGs) among the three cluster patterns using the ssGSEA method. The roles of NET scores in the prediction of immunotherapy were investigated by Immunophenoscores (TCIA database) and validated in two independent cohorts (GSE135222 and IMvigor210). The prediction of targeted drug benefits was implemented using the “pRRophetic” and Gene Set Cancer Analysis (GSCA) datasets. Real-time quantitative reverse transcription polymerase chain reaction (RT-PCR) was performed to identify the reliability of the core genes’ expression in kidney cancer cells.

**Results:**

Three NET-related clusters were identified in the ccRCC cohort. The patients in Cluster A had more metabolism-associated pathways and better overall survival outcomes, whereas the patients in Cluster C had more immune-related pathways, a higher immune score, and a poorer prognosis than those in Cluster B. Based on the DEGs among different subtypes, patients with ccRCC were divided into two gene clusters. These gene clusters demonstrated significantly different immune statuses and clinical features. The NET scores were calculated based on the ten core genes by the Gene Set Variation Analysis (GSVA) package and then divided ccRCC patients into two risk groups. We observed that high NET scores were associated with favorable survival outcomes, which were validated in the E-MTAB-1980 dataset. Moreover, the NET scores were significantly associated with immune cell infiltration, targeted drug response, and immunotherapy benefits. Subsequently, we explored the expression profiles, methylation, mutation, and survival prediction of the 10 core genes in TCGA-KIRC. Though all of them were associated with survival information, only four out of the 10 core genes were differentially expressed genes in tumor samples compared to normal tissues. Finally, RT-PCR showed that MAP7, SLC16A12, and SLC27A2 decreased, while SLC3A1 increased, in cancer cells.

**Conclusion:**

NETs play significant roles in the tumor immune microenvironment of ccRCC. Identifying NET clusters and scores could enhance our understanding of the heterogeneity of ccRCC, thus providing novel insights for precise individual treatment.

## Introduction

Renal cell carcinoma (RCC) is one of the most common urological carcinomas (1). In 2022, the number of tumor cases and cancer-associated deaths in China are expected to reach 7,410 and 46,345, respectively (2). Although the diagnosis and management of RCC have improved (3), its incidence is expected to increase globally. Moreover, approximately 30% of patients are diagnosed with advanced ccRCC, develop distant metastases, and have a poor prognosis due to the atypical symptoms in the early stage of ccRCC (1). ccRCC is the most common subtype of RCC (4). Thus, for better personal precision therapy and management, investigating novel biomarkers is an urgent necessity.

Neutrophils are one type of affluent inflammatory cell in the tumor microenvironment (TME). They could activate cancer cells and desorb modified DNA structures coated with cytoplasmic and granular proteins (5). The web-like structures released by neutrophils to trap microorganisms are termed neutrophil extracellular traps (NETs) (6, 7). Commonly, NETs play critical roles in infectious and non-infectious conditions, such as bacterial and viral infections (5), cystic fibrosis (8), and psoriasis (9). Recently, NETs have been reported to be involved in tumor growth, metastatic spread (10, 11), and immunomodulatory (12). Moreover, NET extrusion exerts a protective effect on the tumor from NK cells and T cells (13). NETs can increase the metastatic potential of circulating tumor cells through augmentation of cell cycle progression (14). Hu et al. reported that NETs could promote the dysfunction of glomerular endothelial cells and pyroptosis in diabetic kidney disease (15). NETs are closely associated with dirty necrosis in RCC (16). Several recent studies have documented the scrutiny of NET-related genes for head and neck squamous cell carcinomas (6), non-small-cell lung cancer (17), and breast cancer (18); however, few studies have focused on the functions of NETs in kidney diseases, particularly kidney cancers. Therefore, it is meaningful to explore new NET-related biomarkers to identify the molecular characteristics of NETs in patients with kidney cancer.

Considering the previous findings, we performed a systemic study on NET-related genes to investigate their roles in the ccRCC cohort. In this study, we first screened the expression, protein–protein network, and prognostic values in the TCGA-KIRC dataset. Based on the expression of NET-related genes, we classified ccRCC patients into three clusters. Patients were further stratified into two gene clusters based on the differentially expressed genes (DEGs) among the three NET subtypes. We further constructed a scoring system to predict overall survival (OS), which may form the basis for research on ccRCC precision treatment.

## Methods

### Data collection and processing

The RNA-sequencing dataset of 534 kidney renal clear cell carcinoma (KIRC) samples, which contained mRNA and clinical and survival data, were acquired from UCSC Xena (http://xena.ucsc.edu/). The GSE29609 dataset, which contained 39 KIRC samples, were downloaded from the GEO database. The mRNA expression levels were transformed from counts to transcripts per kilobase million (TPM) values. The batch effects of the two datasets were eliminated by “ComBat” from the “sva” R package, and principal component analysis (PCA) was performed to demonstrate the before and aftereffects. Finally, 573 samples, 14,074 genes were enrolled into our after-batched cohort. The E-MTAB-1980 dataset, which contained 101 patients with ccRCC, was downloaded from ArrayExpress (https://www.ebi.ac.uk/arrayexpress/).

### Exploration of the genetics and biological significance of NET genes in KIRC

According to previous studies (19–22), we acquired a list of published NET gene sets, which had 69 genes with NET initial biomarkers. The mRNA expression and prognostic values of NETs were based on the TCGA-KIRC dataset. The network of 69 genes was explored based on the GeneMANIA (http://genemania.org/) website.

### Unsupervised clustering analysis

The unsupervised consensus clustering algorithm was applied to assess the variability and stability of clusters based on NET-related and NET subtype-related genes from the ConsensusClusterPlus (23) R package. Then Kaplan–Meier survival analysis was performed to explore the prognosis among different clusters based on the survival (24) and survivor (25) R packages.

### Gene set variation analysis

The 50 terms of the HALLMARK pathway, the Kyoto Encyclopedia of Genes and Genomes (KEGG) pathway, and the Reactome pathway were downloaded from the Molecular Signatures Database (MsigDB, http://software.broadinstitute.org/gsea/msigdb/). Then, function enrichments for different subtypes were performed using the GSVA (26) and ClusterProfiler (27) R packages.

### The immune infiltration landscape of the ccRCC cohort

The StromaScore, ImmuneScore, and ESTIMATEScore were calculated with the “ESTIMATE” R package. The ImmuneScore and StromalScore were the abundance of immune and stromal components, respectively. The ESTIMATEScore was the total values of ImmuneScore and StromalScore. The abundance of 23 kinds of infiltrating immune cells (28) was evaluated using the ssGSEA method from the GSVA (26) R package.

### Calculation of NET score (NET-scores)

According to the mRNA expression of NET subtype-related genes, 94 DEGs were used for further univariate Cox regression analysis. Then the NET score was calculated as an enrichment score (ES) by the ssGSEA method from the GSVA R package based on the top ten genes with P <0.05 samples. The ccRCC cohort was divided into high and low NET score groups based on the optimal cutoff value.

### Prognosis, enrichment analysis, genetic alterations, chemokines, immune exploration, and clinical feature analysis based on NET-scores

The prognosis analysis between the high- and low-NET score groups was tested using the log-rank method. The correspondence among different groups, subtypes, and survival outcomes was shown as Sankey diagrams by the “ggalluvial” R package. The hallmark enrichment analysis between different NET score groups was done using the GSVA R package and genetic alterations by the “maftools” (29) R package. The mRNA expression of chemokines between different NET score groups was displayed using a heatmap. The clinical characteristics of “survival outcomes,” “clinical grade,” “TNM,” and “clinical stage” were selected to demonstrate the discrepancy in the different NET score groups.

### Expression levels of immune checkpoints, immunotherapy response, and drug sensitivity of patients in different NET-score groups

Two immunotherapy-treated cohorts, the IMvigor210 cohort (288 urological tumor patients treated with anti-PDL1) and the GSE135222 cohort (27 lung carcinoma patients treated with anti-PD-1/PD-L1), were collected to explore the immunotherapy response ability of NET scores. The pRRophetic (30) package was implemented to predict the half-maximal inhibitory concentration (IC50) of 138 antitumor agents.

### Online analysis

mRNA expression, single nucleotide variation (SNV), copy number variation (CNV), drug sensitivity, and methylation of genes were analyzed by the GSCA database (http://bioinfo.life.hust.edu.cn/GSCA/#/). The protein levels of core genes in human tumor and non-tumor samples were acquired from the Human Protein Atlas (HPA; https://www.proteinatlas.org/). The oncoplot of genes was explored from cBioportal (https://www.cbioportal.org/).

### Cell culture and RT-PCR

Human normal renal tubular epithelial cells (HK-2) and kidney cells (Caki-1 and 786-O) were purchased from the ATCC company. All cells were cultured in RPMI 1640 as previously described (28). Total RNA from the cultured cells was extracted using the Faster reagent (Invitrogen). Relative gene expression was calculated by Eq. 2^−ΔΔCT^, with GAPDH as an internal control. The primers are as follows:

**MAP7** gene 5’-TCATCATGCCCTACAAAGCTG-3’(sense) and 5’-TGCCAGATGTGAGGAAGAGTA-3’(antisense).
**SLC16A12** gene 5’-TGCTTGCATCTACTGGACTCA-3’(sense) and 5’-TGGCAATAGCTGGAGAGTAACA-3’ (antisense).
**SLC27A2** gene 5’-TGGCGCTCCTTATGGGTAACG-3’(sense) and 5’-CTTGGCAGTATCTCTTCGACAG-3’ (antisense).
**SLC3A1** gene 5’-CAGGAGCCCGACTTCAAGG-3’(sense) and 5’-GAGGGCAATGATGGCTATGGT-3’ (antisense).

### Statistical analysis

All data were analyzed using R software (v4.1.1); a P-value less than 0.05 was considered statistically significant. The “limma” (31) R package was used to perform a difference analysis. The Wilcoxon test was used for data that did not accord with a normal distribution. A t-test was used for normally distributed data. Univariate Cox regression analysis and the Kaplan–Meier method were used to assess the prognostic value of DEGs. The forest plot was achieved by “forestplot” (32) R package. All heatmaps were performed *via* the R “pheatmap” package.

## Results

### Expression and prognostic values of NET-related genes in the TCGA-KIRC

**Figure 1:**
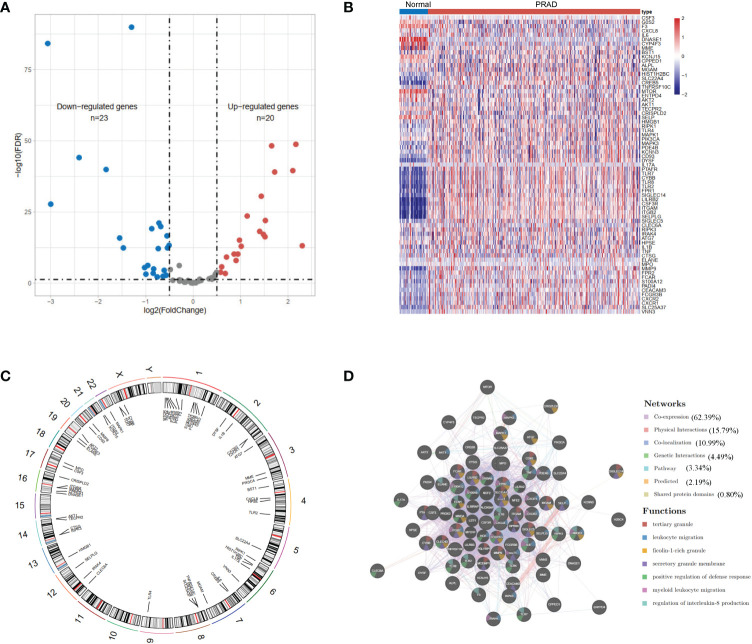
The landscape of neutrophil extracellular trap-associated genes in the TCGA-KIRC. **(A)** Volcano plot and **(B)** heatmap of 69 NET-associated genes in ccRCC and non-tumor samples. **(C)** The location of the NET-associated genes on different chromosomes. **(D)** GeneMANIA gene–gene interaction network showed the correlation among different genes.

We identified 43 differential expression NET-related genes in the TCGA-KIRC dataset, of which 20 are upregulated genes and 23 are downregulated genes with a false discovery rate <0.05 and |log2FoldChange| >0.5 (**Figures 1A, B**, **Supplementary Table 1**). **Figure 1C** shows the locations of the NET-related genes. We then submitted the NET-related genes to GeneMANIA for exploring their interaction network. The results revealed the co-expression to be high (62.39%) and the physical interaction to be 15.79% (**Figure 1D**).

### Identification of NET-related gene subtypes in the ccRCC cohort

**Figure 2:**
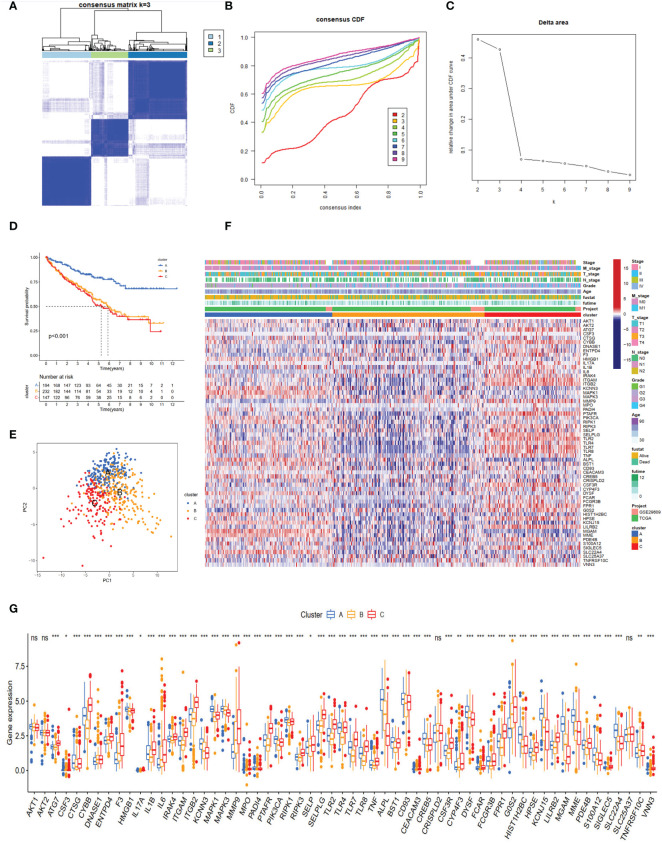
NET subtypes and clinicopathological features of three clusters. **(A)** Consensus matrix of ccRCC samples’ co-occurrence proportion for k = 3. **(B, C)** Consensus clustering CDF for k from 2 to 9. **(D)** The Kaplan–Meier plot showed the overall survival differences among the three subtypes in the ccRCC cohorts. **(E)** Principal component analysis of ccRCC samples grouped by clusters. **(F)** Heatmap showing the association of subtypes with clinical characteristics and expression of neutrophil extracellular trap-associated genes. **(G)** The boxplot of neutrophil extracellular trap-associated genes among different clusters. ns, no significance. *p < 0.05, **p < 0.01, ***p < 0.001.

The TCGA-KIRC and GSE29609 datasets were merged, and PCA demonstrated the before and after batch effects (**Figure S1A**). In the merged ccRCC cohort, we performed unsupervised clustering and classification based on these NET-related genes. Our results showed that k = 3 appeared to be an optimal selection (**Figures 2A–C**). The Kaplan–Meier survival analysis demonstrated that the prognoses of patients were significantly different among these subtypes (log-rank test, P <0.001, **Figure 2D**). Cluster A exhibited better survival better survival advantage than other clusters. The PCA results showed significant differences in NET-related gene expression among the three clusters (**Figure 2E**). The clinicopathological features among the different clusters also revealed significant differences (**Figure 2F**). Moreover, most of the NET-related genes were differentially expressed (**Figure 2G**).

### Characteristics of TME in different subtypes

**Figure 3:**
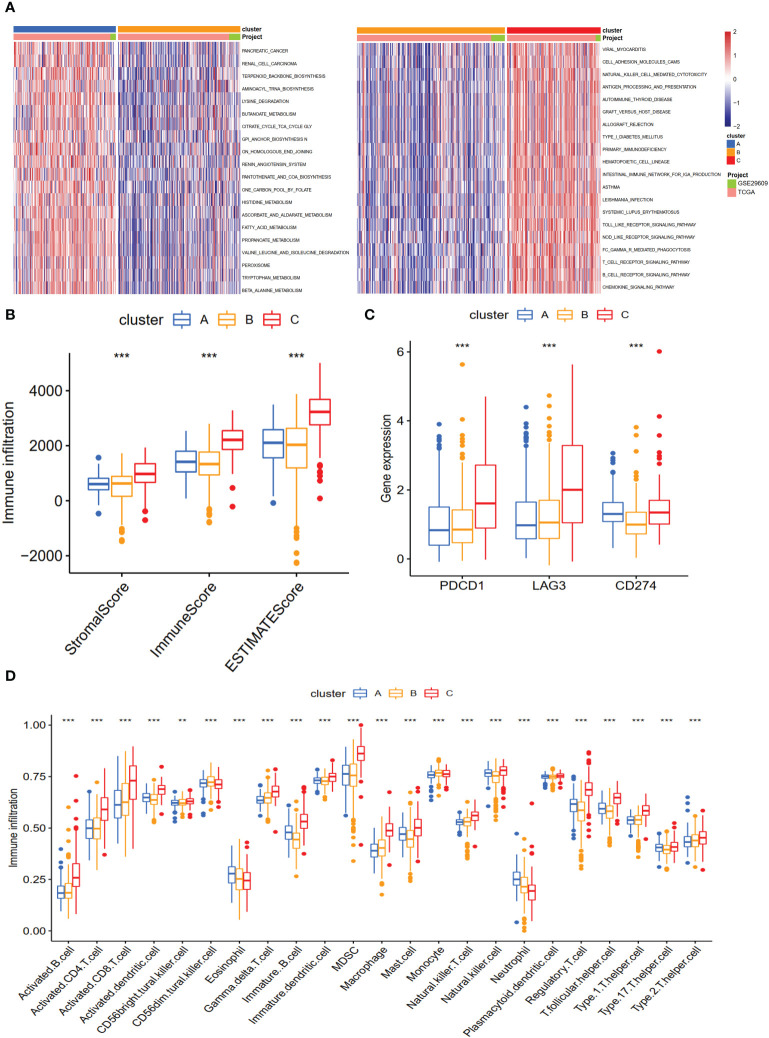
The biological characteristics and landscape of immune status among different subtypes. **(A)** KEGG enrichment analysis of three NET subtypes. **(B)** The ESTIMATE proportion of stromal score, immune score, and ESTIMATE score among the three clusters. **(C)** The gene expression profiles of three common immune checkpoint genes, PDCD1, LAG3, and CD274. **(D)** The infiltration levels of 23 immune cell types among three subtypes. **p < 0.01, ***p < 0.001.

Cluster A was significantly associated with cancer-related and metabolism pathways, such as pancreatic cancer, renal cell carcinoma, butanoate metabolism, histidine metabolism, fatty acid metabolism, tryptophan metabolism, and beta-alanine metabolism (**Figure 3A**). Cluster C was significantly enriched in immune-activated pathways, including NK cell-mediated cytotoxicity, antigen processing and presentation, allograft rejection, autoimmune thyroid disease, T and B cell receptor signaling pathways, and Toll-like and NOD-like receptor signaling pathways (**Figure 3A**). To explore the roles of NET-related genes in the TME of ccRCC, we calculated the TME score using the ESTIMATE method. The results revealed that Cluster C had higher stromal and immune scores than the other two clusters (**Figure 3B**). Analysis of three critical immune checkpoints showed significance among three subtypes (**Figure 3C**). Then, the ssGSEA method was applied to calculate the infiltrating status of immune cells and explore the differential patterns. The results revealed that the infiltration levels of several cells, such as activated B cells, CD4 T cells, and CD8 T cells, were significantly higher in Cluster C than in other clusters (P <0.05, **Figure 3D**), which agreed with the results of the TME score.

### Identification of gene clusters based on DEGs

**Figure 4:**
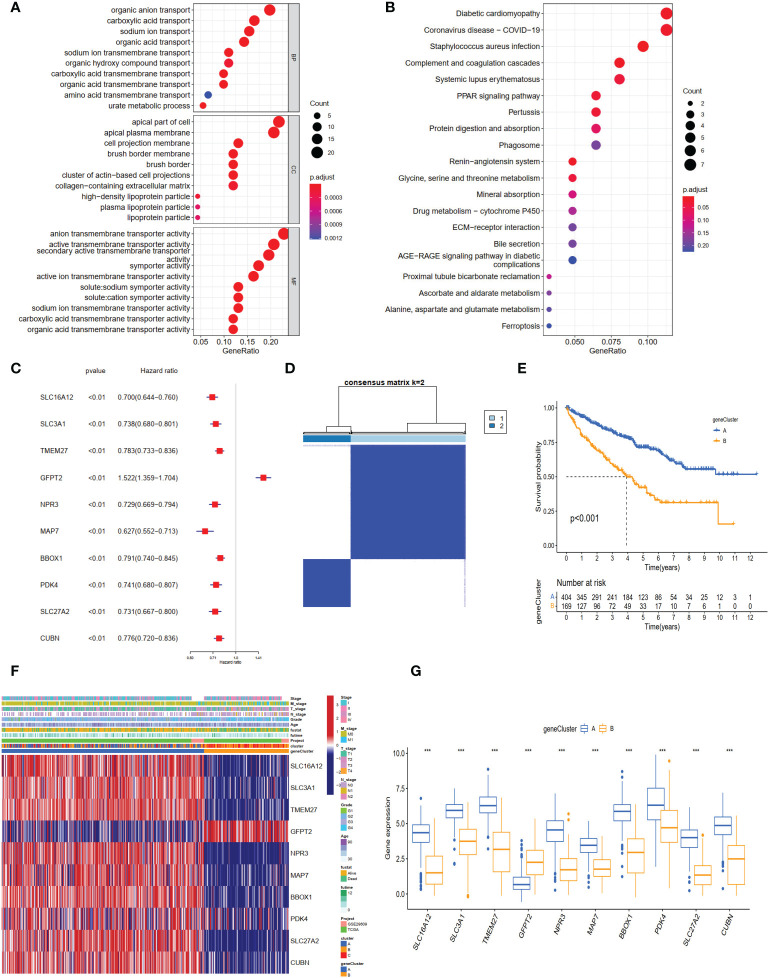
The different expression genes (DEGs), enrichment pathways among different clusters, and consensus clustering based on DEGs. **(A)** The GO and **(B)** KEGG enrichment of different subtypes. **(C)** The forest plot for ten core DEGs based on univariate Cox regression analysis. **(D)** Consensus matrix of ccRCC samples’ co-occurrence proportion for k = 2. **(E)** Kaplan–Meier curves for the two gene clusters of ccRCC patients. The log-rank test shows an overall p <0.001. **(F)** Heatmap showing the relationship among the clinicopathological characteristics of the gene clusters. **(G)** The boxplot of gene expression of ten core genes between the two subtypes. ***p < 0.001.

To explore genes associated with our NET-related clusters, differential gene analyses were performed to select the DEGs among clusters A–C by using “limma” R packages (|logFC| >1.5 and P-value <0.05, **Figure S1B**). The DEGs of these results were then combined, and 94 genes were enrolled for further analysis. The GO enrichment of DEGs demonstrated that the NET subtype-related genes were significantly enriched in transmembrane transport and transporter activity (**Figure 4A**). The KEGG analysis revealed enrichment of immune response-related diseases (such as coronavirus disease 2019 and systemic lupus erythematosus) and cancer-related pathways (**Figure 4B**), which indicated that NETs may play a critical role in immunomodulation. Then, the univariate Cox method was used to explore the prognostic values, and 89 genes were found to be related to OS time (**Supplementary Table 2**). The top ten genes (SLCA16A12, SLC3A1, TMEM27, GFPT2, NPR3, MAP7, BBOX1, PDK4, SLC27A2, and CUBN) with the smallest P-value were selected for further analysis (**Figure 4C**). Based on these 10 prognostic genes, patients were divided into two clusters, namely gene clusters A and B (**Figure 4D**). The Kaplan–Meier curves demonstrated that patients in gene cluster B had poor OS, whereas those in gene cluster A had favorable OS (P-value <0.001, **Figure 4E**). In addition, the gene cluster A patterns were closely related to the late TNM stage (**Figure 4F**). The expression profiles of 10 hub genes were significantly different, consistent with the expected gene clusters (**Figure 4G**).

### Calculation of the NET scores, and evaluation of TME and chemokines in different risk groups

**Figure 5:**
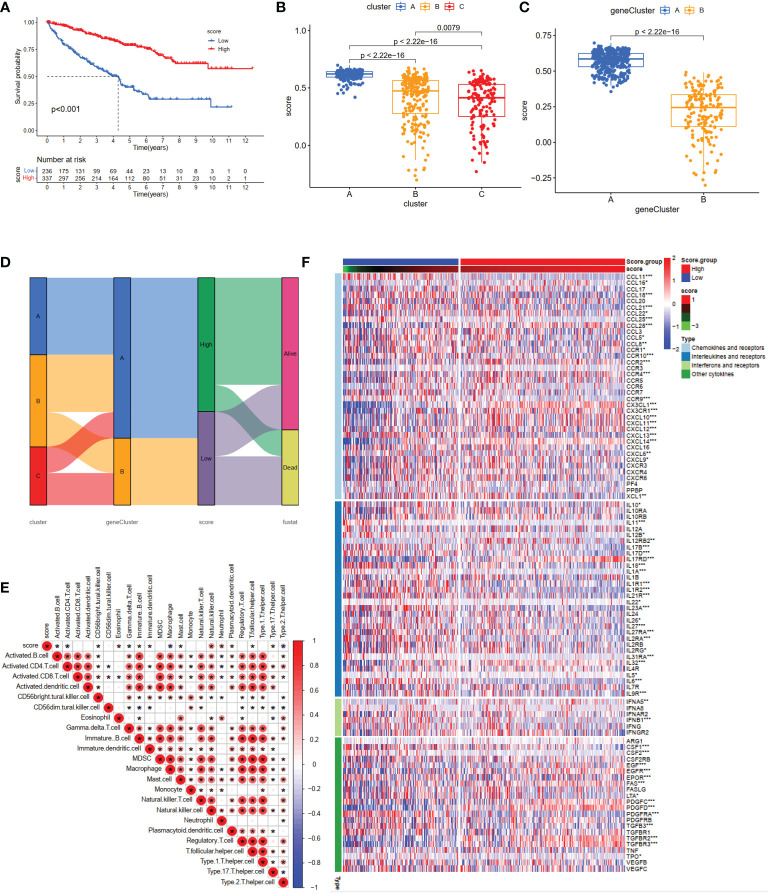
Construction of the NET-score system and clinical prognosis analysis in ccRCC patients. **(A)** Kaplan–Meier curves for high and low NET-score ccRCC patient groups (log-rank test, *P <*0.001). Differences in NET scores among the three clusters **(B)** (P <0.001) and two gene clusters **(C)** (P <0.001). **(D)** Alluvial diagram of NET-associated gene clusters in groups with different gene clusters, NET-score groups, and survival outcomes. **(E)** The correlation matrix of all infiltrating immune cells. Some fractions of immune cells were positively related and are represented in red, whereas others were negatively related and are represented in blue. p <0.05 was the cut-off. **(F)** Heatmap showing the relationship between scoring groups and chemokines, interferons, and cytokines. *p < 0.05, **p < 0.01, ***p < 0.001.

Based on the 10 core genes, we used the ssGSEA method to calculate the NET scores of each patient in the ccRCC cohort. The patients were then divided into high (n = 337) and low (n = 236) risk score groups based on the NET scores. Moreover, compared with the low NET-score group, the high NET-score group had a favorable OS (**Figure 5A**), which was also validated in E-MTAB-1980 (**Figures S1D–G**). We observed a significant difference in the NET scores among different subtypes, which are displayed in **Figures 5B, C**. Cluster C had the lowest NET scores, whereas Cluster A had the highest, revealing that NET scores may be closely associated with immune-infiltration status (**Figure 3B**). **Figure 5D** shows the plots displaying the distribution of patients in three clusters: two gene clusters and two risk score groups.

To investigate the relationship between the abundance of immune cells and NET-scores, we performed the CIBERSORT algorithm to assess. As shown in the correlation matrix, the NET-scores were positive for NK cells and neutrophils, and negative for type 2 helper T cells (**Figure 5E**). The heatmap showed that several chemokines, interleukins, interferons, and their receptors were significantly overexpressed in the high NET-score group (**Figure 5F**), indicating that NET scores may provide novel targets for anti-tumor immunity.

### Clinical characteristics of the NET-scores and functional enrichment between different subtypes

**Figure 6:**
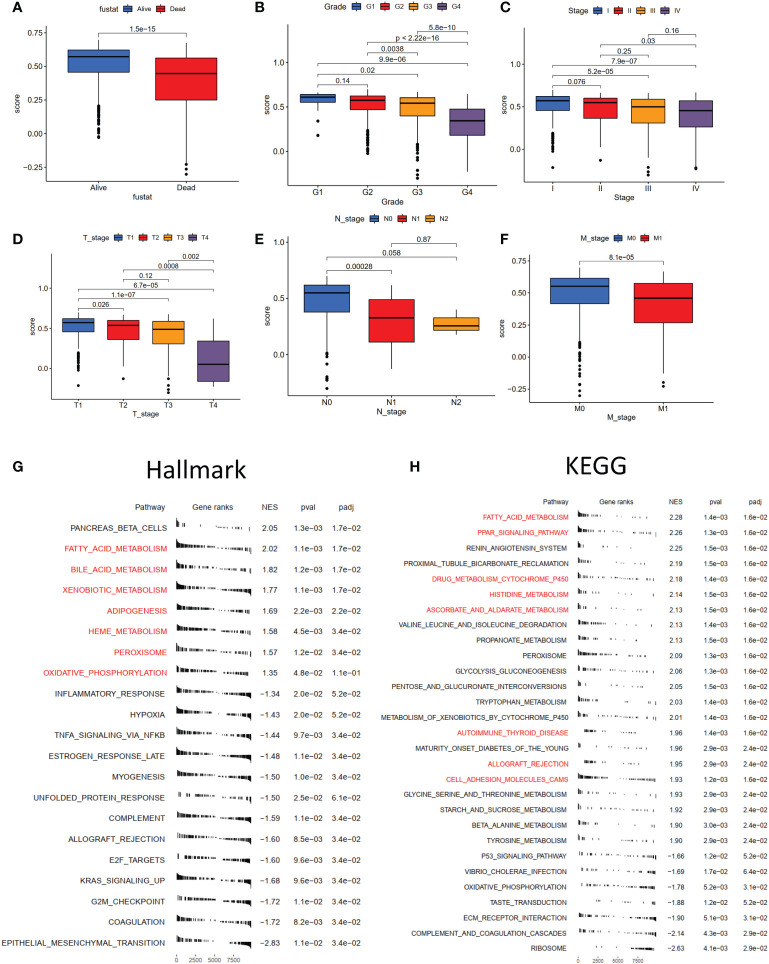
The correlation of NET-scores with clinic-pathological characteristics, hallmark and KEGG enrichment between high- and low-NET-score groups. The boxplot of different survival status **(A)**, clinical grade **(B)**, clinical stage **(C)**, tumor stage **(D)**, regional lymph node status **(E)**, and distant metastasis **(F)**. The hallmark **(G)** and **(H)** KEGG enrichment between high- and low-NET-score groups.

To assess the effect of the NET scores on clinical characteristics, we investigated the association between the NTE scores and several critical features (overall survival status, grade, stage, and TNM stage). The results demonstrated that patients with higher NET scores were associated with a better survival status (**Figure 6A**). Moreover, advanced tumor stages (Grades 3–4, Stages III–IV) also displayed low NET scores (**Figures 6B, C**), which were also observed in tumor size (**Figure 6D**), regional lymph node status (**Figure 6E**), and metastasis (**Figure 6F**).

To further analyze the specific mechanism, common functional enrichments were performed between the high and low NET-score groups using the GSVA method. The hallmark results indicated that high NET scores were associated with several metabolisms and oxidative phosphorylation pathways, such as fatty acid metabolism and xenobiotic metabolism (**Figure 6G**), which were also identified in the KEGG enrichment results (**Figure 6H**). Furthermore, the hallmark and KEGG enrichment showed that the high NET-score group was associated with a series of immune-related pathways, such as allograft rejection and autoimmune thyroid disease (**Figures 6G, H**).

### Evaluation of checkpoints and immunotherapeutic benefit between the high- and low-NET-score groups

**Figure 7:**
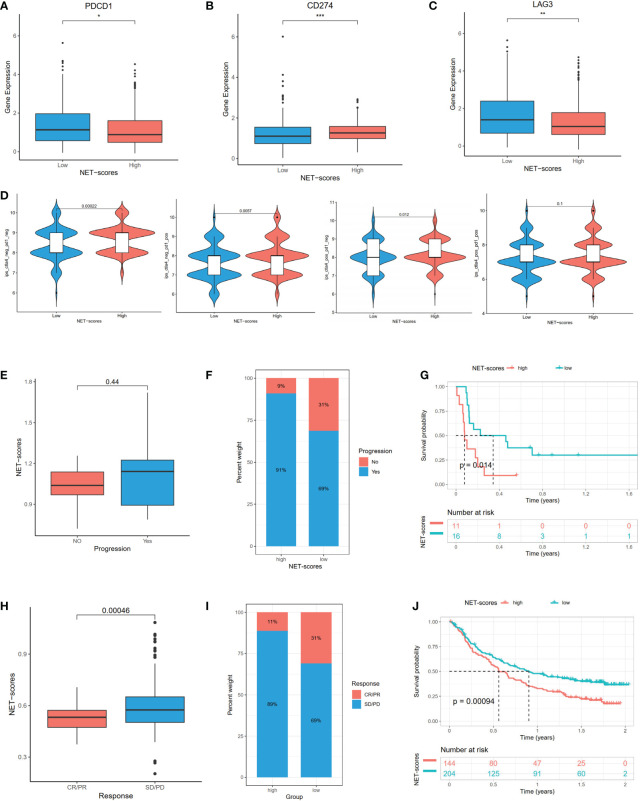
The mRNA expression of immune checkpoint genes and immunotherapeutic benefits. The PDCD1 **(A)**, LAG3 **(B)**, and CD274 **(C)** expression between different NET-score groups. The association between IPS and NET scores **(D)**. The different immunotherapy responses between high- and low-NET-score groups in GSE135222 **(E–G)** and IMvigor210 **(H–J)** datasets. *p < 0.05, **p < 0.01, ***p < 0.001.

We next investigated the expression profiles of three checkpoints (PDCD1, LAG3, and CD274), immunophenoscores (IPS), and immune-checkpoint therapy response. The results demonstrated that PD-1 (PDCD1) and LAG3 were significantly higher in the low NET-score group than the high NET-score group, whereas the PD-L1 (CD274) level displayed a reverse discrepant trend (**Figures 7A–C**). According to the above results, we speculated that the PD-1 inhibitor is more reactive in the low NET-score group and the PDL-1 inhibitor is more effective in the high NET scores. IPS, as the novel method for evaluating the potential clinical efficacy of immunotherapy, was calculated to predict the immunotherapeutic benefit. The results revealed that the high IPS with a positive CTLA-4 signature was associated with high NET-scores (**Figure 7D**).

In the subsequent analysis, we included two public datasets, GSE135222 and IMvigor210 to predict the immunotherapeutic efficacy. Patients with low NET scores were more likely to benefit from immunotherapy (**Figures 7E, H**). Compared to the high-risk group, there was an increase in patients with responses in the low-risk group (**Figures 7F, I**). Patients with low NET scores showed significant immunotherapeutic benefits and favorable survival (**Figures 7G, J**).

### Pathway activity and drug sensitivity analysis

**Figure 8:**
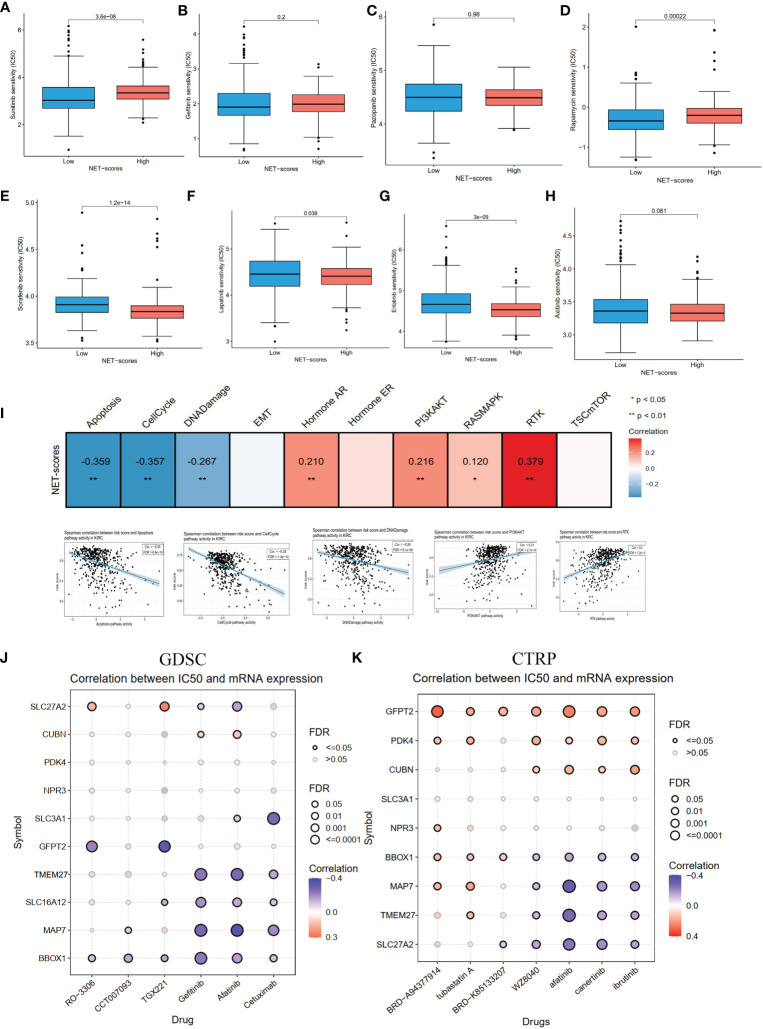
The pathway activity, drug sensitivity in ccRCC cohorts and pan cancer. **(A–H)** The drug sensitivity of eight common targeted compounds. **(I)** The associations of NET scores with activity pathways in the TCGA-KIRC dataset. **(J)** The correlation between gene expression and the sensitivity of GDSC drugs in pan-cancer. **(K)** The correlation between gene expression and the sensitivity of CTRP drugs in pan-cancer.

As chemotherapy is still a traditional therapy method for ccRCC, particularly for advanced ccRCC, we investigated the response of the two NET-score groups to common chemo-drugs. As shown in **Figures 8A–H**, compared with the high NET-score group, sunitinib (P-value = 3.6e−08) and rapamycin (P-value <0.001) showed lower IC50 values in the low NET-score group, whereas sorafenib (P-value = 1.2e−14), lapatinib (P-value= 0.038), erotinib (P-value = 3e−09) and axitinib (P-value =0.081) showed higher values in the low NET-score group, suggesting that patients in the low NET-score group were more likely to respond well to sunitinib, and poorly to sorafenib and axitinib than those in the high NET-score group. Based on the GSCA dataset, we first explored the activity pathways in the TCGA-KIRC. As shown in **Figure 8I**, the NET scores were negatively associated with apoptosis, cell cycle, and DNA damage and positively associated with PI3K/AKT and RTX pathways. This indicated that the NET scores were more likely to play roles in apoptosis and cell cycle by regulating PI3K/AKT and RTX pathways. The drug sensitivity in the pan-cancer analysis of GDSCs and CTRP is shown in **Figures 8J–K**. The results demonstrated that BRD-A96377914, tubastatin A, BRD-K85133207, WZ8040, afatinib, canertinib, ibrutinib, cetuximab, gefitinib, TGX221, CCT007093, and RO-3306 were more likely to function well.

### Genetic mutations of two NET-score groups, landscape, and validation of core genes

To investigate the mutation status between the two NET-score groups, genetic mutations were analyzed using the maftools (29) R package. The results revealed that the high NET-score group had a higher mutation rate than the low NET-score group (70.05% *vs* 58.4%). The top 10 most frequently mutated genes are displayed in **Figure S1C**. Subsequently, the landscape of 10 core genes was explored in the TCGA-KIRC. The results demonstrated that only four genes (MAP7, SLC16A12, SLC27A2, and SLC3A1) were DEGs in ccRCC compared to normal samples (**Figure S2A**). Four genes had more than a 1% mutation rate (**Figure S2B**). The 10 core genes were significantly associated with DSS, OS, and PFS (**Figure S2C**). Several genes were positively correlated with methylation levels, whereas CUBN, MAP7, and SLC16A12 were closely associated with copy number variation (CNV) levels (**Figure S2D**). Most of the genes (9/10) were positively associated with PI3K/AKT, RTK, and hormone AR activity and negatively associated with apoptosis, cell cycle, and DNA damage (**Figure S2E**). Considering only four genes were DEGs, we explored these four genes in the CCLE dataset. The results revealed that the basal expression profiles of MAP7, SLC16A12, and SLC3A1 were high in kidney cancer cells (**Figure S2F**). The RT-PCR showed that MAP7, SLC16A12, and SLC27A2 were decreased in 786-0 and Caki-1 compared with HK2, while SLC3A1 increased (**Figure S2G**), which was consistent with the results of the TCGA-KIRC (**Figure S2A**). The protein levels of HPA demonstrated that MAP7 and SLC27A2 levels were lower, and SLC3A1 levels were higher, in tumor tissues than in normal samples (**Figure S2H**), in accordance with the results of the TCGA-KIRC and RT-PCR.

## Discussion

ccRCC, the most common subtype of RCC, is highly associated with poor clinical outcomes (33). Emerging treatments such as targeted drugs and immunotherapy have significantly enhanced the prognosis of patients with advanced ccRCC; however, the effectiveness of these treatment strategies still needs to be improved (34). Moreover, ccRCC has strong immune-associated characteristics (35). Thus, reliable biomarkers are urgently required to predict recurrence risk and guide treatments. NETs and immune cell infiltrations have been reported to have critical roles in tumor progression (36). Sivan et al. first described the association between NETs and cancer (Ewing sarcoma) (12). Subsequently, there are increasing studies on NETs and cancer. For example, NETs drive the process of endothelial-to-mesenchymal transition (37). Aldabbous et al. identified that NETs promote angiogenesis (38). Moreover, NETs promote cancer-associated thrombosis *via* thrombin generation and the conversion of fibrinogen to fibrin (39). Additionally, many prognostic signatures based on NETs have been reported in human cancers (19, 20). However, whether NETs are also involved in tumor prognosis and play prognostic values in ccRCC has not been explored. Therefore, we collected the expression profiles of NET-related genes and clinical characteristics from the TCGA, GEO, and ArrayExpress datasets and comprehensively explored the NET-related genes in the ccRCC cohort.

In the current study, we first examined the roles of NET-related genes in the TCGA-KIRC and found that 43 of 69 genes were significantly differentially expressed in the tumor samples compared to non-tumor tissues. Moreover, most of the genes were prognostic genes. Then, three NET-related subtypes (Clusters A–C) were identified in the ccRCC cohort by consensus cluster algorithms. It was found Cluster B had low levels of NET-related genes and low abundance of immune cells infiltration, whereas Cluster C had high levels of NET-related genes and immune cell infiltration. Moreover, the three subtypes had significantly different overall survival outcomes. The differences in mRNA expression profiles among the three subtypes were dramatically correlated with metabolism- and immune-related biological pathways. We identified two gene clusters, A and B, based on the DEGs among the three NET-related subtypes. Our findings suggested that NETs act as a predictor for clinical survival outcomes, targeted drugs, and the immunotherapy response of ccRCC. Therefore, we established the NET scores based on 10 hub genes by using the ssGSEA method. Patients with low and high NET scores showed significant discrepancies in clinical characteristics, prognosis, immune cell infiltrations, immune checkpoints, and activity signal pathways.

As for the 10 core genes, MAP7, SLC16A12, SLC27A2, and SLC3A1 were significantly different in patients with ccRCC when compared to non-tumor samples. MAP7, Microtubule-associated protein 7, functions as a regulator of microtubule bundling and dynamics. Several studies had reported MAP7 involved in cell cycle progression (40) and autophagy pathway in cancers (41). SLC16A12, SLC27A2, and SLC3A1 belonged to the solute carrier group of membrane transport proteins (42). Liu et al. reported that decreased expression of SLC16A12 mRNA levels was associated with a poor prognosis for ccRCC (43). Upregulation of SLC27A2 could inhibit the proliferation and invasion of RCC *via* a CDK3-mediated pathway (44). SLC3A1, the cysteine carrier, has been reported to promote breast cancer tumorigenesis *via* AKT signaling (45). In our study, we found MAP7, SLC16A12, and SLC27A2 in kidney cancer cells when compared with normal kidney cells, which agreed with the results of the TCGA-KIRC. Generally, the results indicated that MAP7, SLC16A12, SLC27A2, and SLC3A1 could be the biomarkers for the complement system of ccRCC.

Immunotherapies, particularly immune checkpoint inhibitors (ICIs), have transformed the treatment of several advanced carcinomas (46–49). Although clinical benefits have been achieved when patients with ccRCC receive ICIs, the responses demonstrated personal heterogeneity (50). Thus, looking for markers to predict the responses of ICI treatment is highly important. In our study, we observed higher expression levels of PD1 and LAG3 in Cluster C and low NET scores. Moreover, we found that the NET scores were significantly lower in patients responding to ICIs, which identified their predictive effects. These results suggested that patients with low NET scores and higher expression levels of PD1 and LAG3 are more likely to respond to ICI treatment. Considering that targeted therapy remains the recommended treatment for patients with advanced ccRCC, we evaluated eight common drugs based on the GDSC dataset. The results showed that a low-NET-score group might be likely to acquire benefits from sorafenib, axitinib, gemcitabine, and lapatinib treatments. The above results indirectly suggested the use of NET modifications for predicting clinical benefits from ICI and targeted therapy.

Although in the present study we identified three NET clusters, established a NET-score system, and provided a novel perspective for precise immunotherapy and targeted therapy for ccRCC, several limitations should be addressed. First, all analyses were performed on data obtained from public datasets; thus, the analysis results might be influenced by an intrinsic case selection bias. Large-scale prospective studies and cell and animal experimental research are necessary to confirm our findings.

In conclusion, our study expansively displayed the relationship between NET modification patterns and TME, clinical characteristics, and prognosis. We also assessed the treatment sensitivity prediction of NETs in ICI and targeted treatments. Finally, we constructed a NET-score system for quantifying the NET patterns of patients with ccRCC and validated the expression of core genes. Thus, the findings of the present study might facilitate our understanding of ccRCC and provide ideal strategies for individual treatment.

## Data availability statement

The original contributions presented in the study are included in the article/**Supplementary Material**. Further inquiries can be directed to the corresponding author.

## Author contributions

Y-PZ and Z-HT designed the study. Z-HT and W-CL performed the experiment and data analysis. Z-CL, Y-XW, and Z-WH conducted the Q-PCR. Z-WH and Z-HT contributed to manuscript writing, reviewing, and revision. Y-PZ and Z-HT supervised the study. All authors contributed to the article and approved the submitted version.

## Supplementary material

Supplementary Figure 1 The PCA differential, mutation analysis in different groups, and validation of E-MTAB-1980. **(A)** Principal component analysis of before and after removing batch effects in TCGA-KIRC and GSE29609 cohorts. **(B)** The volcano plot for differential analysis between different clusters. **(C)** The waterfall plot for high and low NET-scores in TCGA-KIRC. **(D)** Consensus matrix, **(E)** consensus clustering CDF, and **(F-G)** Kaplan–Meier curves for different clusters, and NET scores for ccRCC samples in E-MTAB-1980.

Supplementary Figure 2 The expression profiles, mutation, and survival analysis of cores genes. The differential analysis **(A)**, mutation **(B)**, survival analysis **(C)**, correlations between mRNA expression and CNV, methylation **(D)**, pathways activity **(E)** based on TCGA-KIRC dataset. The mRNA expression of four differential expressed cores in CCLE dataset (F). RT-PCR validated the mRNA expression of four differential expressed cores **(G)**. The expression profiles of differential expressed cores in HPA dataset. Notes: SLC16A12 cannot be found in HPA.

Supplementary Table 1 The differential expression results of NET-related genes in TCGA-KIRC dataset.

Supplementary Table 2 The results of univariate Cox regression for 94 DEGs in ccRCC cohort.
