# Supplementary figures and images for: Neutrophil extracellular traps-associated modification patterns depict the tumor microenvironment, precision immunotherapy, and prognosis of clear cell renal cell carcinoma
Source: Front Oncol. 2022 Dec 22;12:1094248. doi: 10.3389/fonc.2022.1094248 (PMC9813599; doi:10.3389/fonc.2022.1094248)

Figure S1

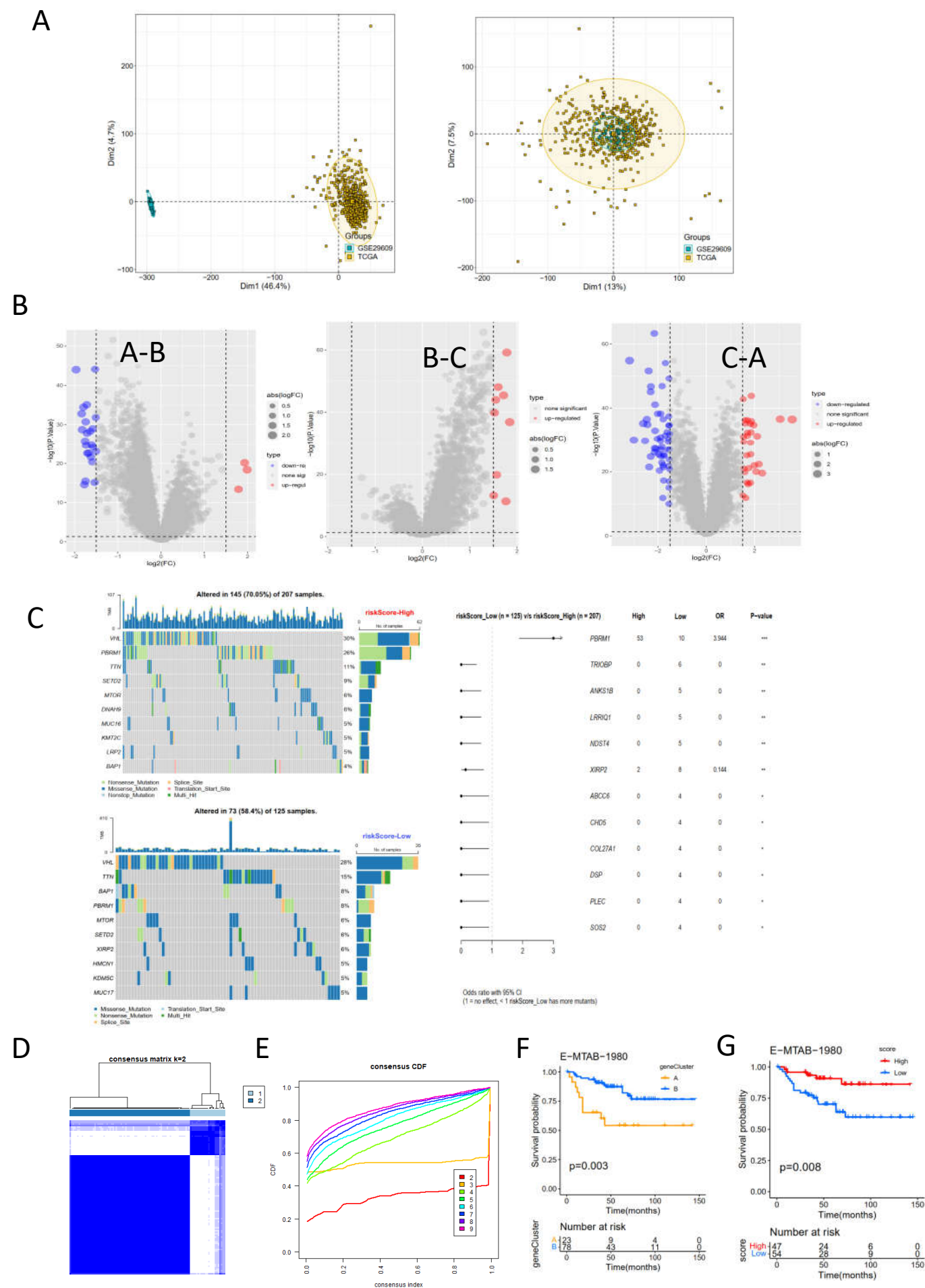

Supplement: Supplementary Figure 1 — The PCA differential, mutation analysis in different groups, and validation of E-MTAB-1980. (A) Principal component analysis of before and after removing batch effects in TCGA-KIRC and GSE29609 cohorts. (B) The volcano plot for differential analysis between different clusters. (C) The waterfall plot for high and low NET-scores in TCGA-KIRC. (D) Consensus matrix, (E) consensus clustering CDF, and (F-G) Kaplan–Meier curves for different clusters, and NET scores for ccRCC samples in E-MTAB-1980. [file Image_1.pdf]

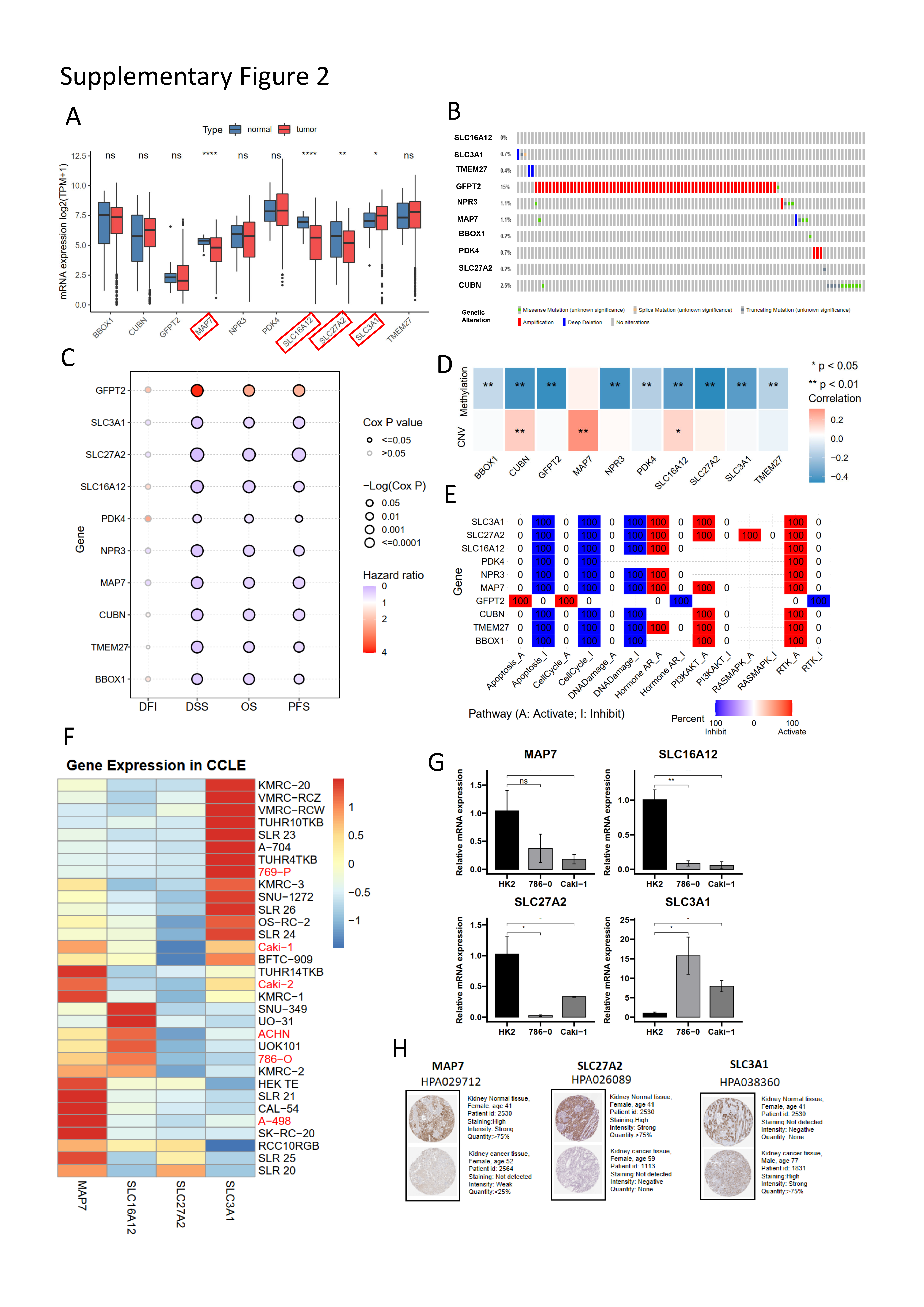

Supplement: Supplementary Figure 2 — The expression profiles, mutation, and survival analysis of cores genes. The differential analysis (A), mutation (B), survival analysis (C), correlations between mRNA expression and CNV, methylation (D), pathways activity (E) based on TCGA-KIRC dataset. The mRNA expression of four differential expressed cores in CCLE dataset (F). RT-PCR validated the mRNA expression of four differential expressed cores (G). The expression profiles of differential expressed cores in HPA dataset. Notes: SLC16A12 cannot be found in HPA. [file Image_2.tiff]
